# Effects of Ionizing Radiation on *Apis mellifera* L. Queens †

**DOI:** 10.3390/toxics13121057

**Published:** 2025-12-05

**Authors:** Margot Crevet, Béatrice Gagnaire, Luc P. Belzunces, Nicolas Dubourg, Guillaume Kairo, Gianni Marcuccini, Michel Pélissier, Jean-Luc Brunet

**Affiliations:** 1 Autorité de Sûreté Nucléaire et de Radioprotection, Laboratoire d’Ecologie et d’Écotoxicologie des radionucléides, 13115 Saint Paul lez Durance, France; 2 INRAE, UR 406 Abeilles & Environnement, Toxicologie Environnementale, 84914 Avignon, France; 3 Association pour le Développement de l’APIculture provençale, 13100 Avignon, France

**Keywords:** ionizing radiation, *Apis mellifera* L., honeybee, queen, fertility, biomarkers

## Abstract

In honeybees (*Apis mellifera* L.), the queen ensures the reproduction of the colony. Environmental contamination that could alter this function could compromise the survival of the colony. Ionizing radiation could be such a factor, but its effects have never been studied in queens. The effects of gamma irradiation on queen bees were evaluated under laboratory conditions. The queens were irradiated for 14 days at dose rates of 0.1, 13, and 3500 µGy/h, and mortality, reproductive potential, and several physiological biomarkers were investigated. Irradiation did not affect the survival of the queens or the number of sperm stored in the spermatheca. However, sperm viability and reproductive potential decreased significantly at 13 and 3500 µGy/h. Among the biomarkers analyzed (antioxidant defenses, detoxification, metabolism, immunity, neural activity), no significant differences were observed between the modalities, with the exception of an increase in thoracic LDH activity at 13 µGy/h, confirmed by multivariate analyses indicating metabolic changes. These results show that ionizing radiation does not induce lethality at the tested dose rates, but significantly impaired fertility and metabolism of queen bees, with potential consequences for colony stability, whose ecological significance remains to be confirmed under field conditions.

## 1. Introduction

The honeybee (*Apis mellifera* L.) is a key pollinator in various ecosystems. It plays a decisive role in the sexual reproduction of many plant species, including both agricultural crops and wild plants, thereby contributing to the maintenance of biodiversity and the sustainability of food production [1]. However, honeybee colonies are experiencing a marked decline worldwide, posing a major threat to pollination services and the beekeeping industry [2]. Reproduction is one of the essential pillars ensuring the persistence of bee colonies. In *A. mellifera* L., the queen is the only reproductive female in the colony. She lays fertilized and unfertilized eggs that will develop into workers and males, respectively [3]. The workers perform all the tasks necessary for the colony’s development and survival, while the males have the main role of reproduction during mating flights with new queens [3,4]. The formation of new queens in the colony can result from several processes: supersedure, triggered by a decline in the queen’s reproductive performance or pheromone signals; requeening, following the accidental or natural disappearance of the queen; or swarming, when the queen leaves with some of the workers to found a new colony [3,5]. The workers build queen cells provided with the egg of the old queen, which will develop into a new queen after about 16 days [6]. About seven to ten days after emerging, the young queen makes one to three nuptial flights during which she mates with about ten males per flight [3]. Following mating, the queen stores the sperm in the spermatheca, an organ that ensures the preservation of the sperm over time. A newly fertilized queen can store up to ten million spermatozoa [7], ensuring continuous egg-laying capacity over a period of three to five years [3]. During oviposition, a small amount of sperm is released from the spermatheca and migrates to the median oviduct, where fertilization of the oocytes takes place [8]. This highly regulated process enables the production of diploid eggs, which will become females, ensuring the colony’s survival. The spermatheca is therefore a key organ in bee reproduction. Certain pollutants, particularly pesticides, can alter the fertility and reproductive capacity of queens. For example, thiamethoxam as well as Boscalid reduce the number of spermatozoa in the spermatheca [9,10]. A decrease in the viability of spermatozoa reduces the health of queens and is closely linked to an increase in colony mortality [11].

Reproduction can also be affected by environmental contamination through ionizing radiation, whether from nuclear activities or accidental releases, as observed in certain insects, including *Culex pipiens* [12]. In this context, eusocial bees have been designated by the International Commission on Radiological Protection (ICRP) as Reference Animal and Plant (RAP), a representative organism used to assess and interpret the potential effects of ionizing radiation on a broader taxonomic or ecological group. For the bee RAP, the Derived Consideration Reference Level (DCRL)—defined as the range of dose rates within which there is a probability of some deleterious effects of ionizing radiation occurring to individuals of that type—has been set in the range of 400 to 4000 µGy/h. This range was established by inference from other invertebrates, as no specific experimental data were available for hymenoptera [13]. However, negative effects from ionizing radiation on the reproduction and growth of bumblebee colonies, *Bombus terrestris audax*, have since been demonstrated with a 30–45% reduction in reproduction for an exposure of 100 μGy/h, below the lowest value of the DCRL [14].

This study aims to evaluate, under laboratory conditions, the effects of exposure to ionizing radiation on the reproductive potential of a honeybee colony. In this context, the study focuses specifically on the impact of gamma irradiation on already fertilized queens. The objectives of this study are to (i) assess the impact of ionizing radiation on bee queen mortality and fertility by examining their reproductive potential and (ii) analyze the effects of radiation on the main physiological functions of queen bees, in particular defenses against oxidative stress, immune response, and energy metabolism, through the use of physiological markers. To this end, queen bees were irradiated at dose rates of 0.1 (control), 13, and 3500 µGy/h. The dose rate of 13 µGy/h is below the lower bound of the DCRL range defined by the ICRP for bees (400 µGy/h) [13], within which no significant adverse effects are expected in insects. This dose rate is also slightly above the threshold of 10 µGy/h proposed by Garnier-Laplace et al. [15], below which 95% of ecosystems are considered to be protected from the effects of ionizing radiation. The higher dose rate of 3500 µGy/h was chosen in order to examine the mechanisms of action at exposure levels close to the upper bound of the DCRL range defined by the ICRP for bees (4000 µGy/h), within which adverse biological effects may start to occur.

## 2. Materials and Methods

### 2.1. Apis mellifera L. Queens

Young fertilized Apis mellifera queens were provided by Mélodie Perrin, an organic breeder (Arles, France). They were approximately one month old at the start of the experiment. All of them were naturally fertilized at the same time and under the same conditions when they were 7 to 10 days old. They were then individually placed in cages (5.5 × 4.5 × 7.5 cm) with approximately 30 female worker honeybees. To obtain the accompanying honeybees, three frames of capped brood were taken from INRAE hives (Avignon, France) and placed in an incubator at 32 °C ± 2 °C and a relative humidity of 60 ± 5% for approximately 72 h. After emergence, the honeybees were homogenized before being distributed in cages. The queens originated from three distinct genetic lines and were evenly distributed across all experimental modalities. The honeybees were fed a sucrose syrup (70% (*w*/*v*) sucrose) administered via a 5 mL perforated tube per cage, replaced every 2 to 3 days. A feeder containing crushed pollen was also placed in each cage for the first three days of the experiment. Filter paper sheets were placed at the bottom of each cage and replaced every 2–3 days. Dead workers or queens were removed and counted at the same frequency.

### 2.2. Irradiation in Laboratory Conditions

Queens were exposed to gamma radiation from a [^137^Cs]-Cesium source for a period of 14 days using the MICADO’ Lab irradiator (ASNR, Cadarache, France). An irradiation duration beyond 14 days was not considered, as maintaining queens in cages without a colony for extended periods leads to a high risk of mortality. The cages were placed in climatic chambers maintained at 28 ± 2 °C with a relative humidity of 60 ± 5%. Glass RPL microdosimeters (GD-300 series, Chiyoda Technologies, Tokyo, Japan) were attached to each cage to measure the radiation dose received by the queens. To ensure homogeneous dose rate distribution, the cages were regularly repositioned within the chamber by rolling. Three irradiation conditions were set up. Some queens were not irradiated and were placed in an environment separate from the irradiator, where they were exposed only to an ambient dose rate of 0.1 µGy/h (C). Others were subjected to a low-dose irradiation of 13 µGy/h (L), while the last group was exposed to a high dose rate irradiation of 3500 µGy/h (H). A total of 94 queens were used for analyses (*n* = 32 (C), *n* = 31 (L, H)). To organize dissections efficiently, queens were divided into four groups. Irradiation for each group started one day apart, ensuring a consistent and manageable number of dissections per day.

### 2.3. Reproductive Potential

After 14 days of irradiation, the queens were anesthetized using CO_2_, then their spermathecae were collected in order to assess the effect of ionizing radiation on sperm count and viability, based on the protocol of Kairo et al. [16]. After collection, each spermatheca was ground in PBS buffer. For sperm count, the spermathecal homogenate was diluted 1/1500 in PBS buffer before counting spermatozoa using a Neubauer Petroff counting chamber, where six homogenate deposits were made. An image of the central grid was acquired for each deposit at 400× using a light microscope with a camera (Advanced CMOS Camera) and image capture software (ToupView version 3.7, Advilab, Allauch, France). Spermatozoa were then counted on these images, and counts from the six deposits were averaged for each queen. Sperm viability was assessed using the eosin–negrosin staining method [17] with the Vita-Eosin kit (RAL Diagnostics). The spermathecal homogenate diluted in PBS buffer was mixed with eosin to stain dead spermatozoa pink. After 5 min of incubation, negrosin was added to generate a dark contrast, allowing for better distinction between live and dead spermatozoa. Three cell smears per queen were made on microscope slides from spermatozoa suspension. The analysis was then carried out under a light microscope (×200), counting 100 spermatozoa per slide and differentiating live cells from dead cells to establish a viability ratio. The reproductive potential was calculated by multiplying the total number of spermatozoa per µL of sperm by their viability rate, thus making it possible to assess the impact of ionizing radiation on queen fertility. The remainder of the spermathecal homogenate was frozen at −20 °C for subsequent integrity analysis.

### 2.4. Biomarker Analysis

After removal of the spermatheca, the head, abdomen, and thorax were separated for physiological analyses. Tissues were individually placed in tubes, weighed, and stored at −20 °C. Tissues were homogenized using a TissueLyser II high-speed homogenizer (Qiagen, Les Ulis, France) for heads and Precellys Evolution for thoraxes and abdomens. Homogenization was performed in five 10 s cycles, with 30 s intervals, after the addition of an extraction medium composed of 10 mM sodium chloride, 1% (*w*/*v*) Triton X-100, 40 mM sodium phosphate (pH 7.4) and a mixture of protease inhibitors constisting of 2 μg/mL pepstatin A, leupeptin and aprotinin, and 0.1 mg/mL soybean trypsin inhibitor. This preparation allowed obtaining 10% (*w*/*v*) tissue extracts, according to the protocol of Belzunces et al. [18]. After homogenization, the extracts were centrifuged at 4 °C for 20 min at 15,000× *g*, and the supernatants were kept on ice before biochemical analyses. Analyses were also performed on the remaining spermathecal samples after assessment of the reproductive potential. The residual supernatant from the spermathecal samples was rinsed with PBS buffer and then centrifuged for 20 min at 15,000× *g*. The supernatant was discarded, and the pellet was suspended in PBS and then centrifuged a second time under the same conditions to remove the remaining supernatant. Only the pellet containing the spermatozoa was kept aside. For physiological analyses, each sample (tissue and spermatozoa extract) was analyzed in triplicate using a UV–visible microplate spectrophotometer (Biotek, Colmar, France).

The use of multiple biomarkers aimed to investigate a broad spectrum of physiological traits in order to achieve a comprehensive understanding of the potential effects of ionizing radiation on antioxidant, detoxification, metabolic, and immune functions (Table 1). Acetylcholinesterase (AChE) was assayed at 412 nm in a reaction medium containing 1.5 mM 5,5′-dithiobis-2-nitrobenzoic acid (DTNB), 0.3 mM acetylcholine iodide (AcSCh.I), and 100 mM sodium phosphate at pH 7.0, according to the method of Ellman et al. [19], modified by Belzunces et al. [18]. Malondialdehyde (MDA) was determined using the TBARS Assay Kit (Cayman) at 530 nm, following the method described by Abdelkader et al. [20]. Catalase (CAT) was assayed by hydrogen peroxide (H_2_O_2_) decomposition at 240 nm. The reaction medium contained 30 mM H_2_O_2_ and 100 mM sodium phosphate at pH 7.0, according to the protocol of Aebi [21]. Superoxide dismutase (SOD) was indirectly measured by the generation of reduced nitro blue tetrazolium (NBT), which was monitored at 560 nm. The reaction medium contained 0.1 mM EDTA, 0.1 mM xanthine, 0.025 mM NBT, 8.33 mU/mL xanthine oxidase, and 50 mM phosphate/carbonate at pH 7.8, according to the protocol of Boldyrev et al. [22]. Total antioxidant capacity was determined at 405 nm using the Antioxidant Assay Kit (Cayman Chemical), following the method described by Williams et al. [23]. Glucose-6-phosphate dehydrogenase (G6PDH) was determined by monitoring the formation of reduced nicotinamide adenine dinucleotide phosphate (NADPH) at 340 nm. The reaction medium contained 10 mM magnesium chloride (MgCl_2_), 1 mM glucose-6-phosphate (G6P), 0.5 mM nicotinamide adenine dinucleotide (NADP^+^), and 100 mM Tris-HCl at pH 7.4, according to the protocol of Renzi et al. [24]. Glyceraldehyde-3-phosphate dehydrogenase (GaPDH) was measured by the oxidation of reduced nicotinamide adenine dinucleotide (NADH), followed at 340 nm. The reaction medium contains 7 mM 3-phosphoglyceric acid (3-PGA), 4 mM L-cysteine–HCl neutralized with sodium bicarbonate (NaHCO_3_), 2 mM magnesium sulfate (MgSO_4_), 120 µM NADH, 1.2 mM adenosine triphosphate (ATP), 1 mM disodium salt of ethylenediaminetetraacetic acid (EDTA), and 5 units.mL^−1^ 3-phosphoglycerate kinase (3-PGK) and 80 mM triethanolamine at pH 7.0, according to the protocol by Renzi et al. [24]. ATP was determined using the ATPlite kit (Revvity) based on luminescence measurements generated by the oxidation of D-luciferin by luciferase, following the method described by Cree et al. [25]. Lactate dehydrogenase (LDH) activity was determined by regenerating nicotinamide adenine dinucleotide (NAD^+^), followed at 340 nm. The reaction medium contained 5 mM EDTA, 0.2 mM reduced nicotinamide adenine dinucleotide (NADH), 2 mM sodium pyruvate, and 50 mM triethanolamine at pH 7.5, according to the protocol of Al-Lawati et al. [26]. Triglyceride (TG) was determined using the Triglycerides Colorimetric Assay Cayman kit (Bertin). Absorbance was measured at a wavelength of 550 nm. Carboxylesterase 1 (CaE1) was measured at 568 nm in a medium containing 0.0110 mM µM acetylcholinesterase inhibitor (BW284C51), 0.1 mM α-naphthyl acetate (α-NA), and 100 mM sodium phosphate at pH 7.0. The catalysis occurred over 1 min and was stopped by adding 0.2 reaction volumes of a solution containing 10% sodium dodecyl sulfate (SDS) and 4 mg·mL^−1^ fast garnet GBC sulfate salt, following the method described by Gomori et al. [27]. Phenol oxidase (POx) was determined by monitoring the conversion of 3,4-dihydroxy-L-dihydroxyphenylalanine (L-DOPA) to melanin at 490 nm. The reaction medium contained 20 mM sodium chloride (NaCl), 0.4 mg/mL^−1^ L-DOPA, and 10 mM sodium phosphate at pH 7.2, according to the protocol of Alaux et al. [28]. All products used for physiological analyses come from Merck (Saint-Quentin-Fallavier, France) or Bertin Technologies (Montigny-le-Bretonneux, France).

### 2.5. Statistical Tests

Statistical analyses were performed using R (version 4.4.2). Normality was assessed using the Shapiro-Wilk test (stats package) and homogeneity of variances using Levene’s test (car package [29]). When necessary, a Box–Cox transformation (MASS package) was applied. Comparisons between groups were performed using one-way ANOVA, followed by Tukey’s post hoc tests. When normality conditions were not met despite the Box–Cox transformation, a Kruskal–Wallis test (stats package) was applied. Graphical representations were produced with ggplot2 [30], ggpubr [31], and dplyr [30]. Significance letters were generated using the multcompView package [32] and added to the graphical representations. Multimarker analyses were conducted on biomarkers expressed as untransformed relative values, after imputation by variable median and then z-score standardization. The multivariate structure was visualized by discriminant factor analysis (MASS package) with 95% confidence ellipses (ggplot2 [33] and patchwork [34] package), and the importance of the markers was summarized by the loading coefficients (top 10). Overall differences between conditions were tested by PERMANOVA (vegan package [35]) using the Euclidean distance calculated on the standardized data with 999 permutations. When the global PERMANOVA was significant, pairwise PERMANOVA comparisons were performed between conditions to identify which groups differed. In addition, the homogeneity of multivariate dispersion was assessed using betadisper (vegan package [35]), and pairwise differences in dispersion were tested with Tukey HSD post hoc comparisons.

## 3. Results

### 3.1. Effect of Ionizing Radiation on Mortality and Fertility

The effects of ionizing radiation were assessed by monitoring the mortality of queen bees throughout the irradiation period. No mortality was observed under any modality after 14 days of exposure. No significant effect on mortality was found in this study (Table S1).

**Figure 1:**
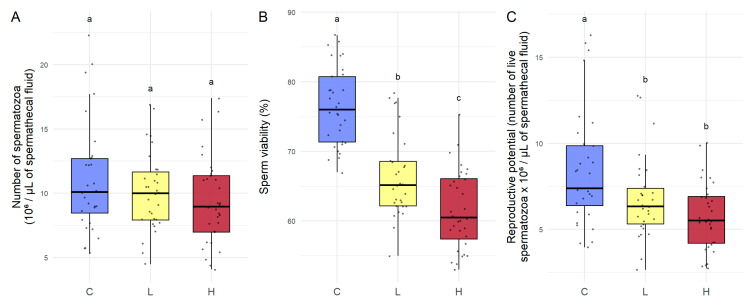
Effects on fertility after 14 days of irradiation. *Apis mellifera* queens irradiated for 14 days with gamma radiation: C (control, non-irradiated), L (irradiated at the low dose of 13 µGy/h), and H (irradiated at the high dose of 3500 µGy/h): (**A**) number of spermatozoa, (**B**) sperm viability, and (**C**) reproductive potential. Data are presented as box plots (*n* = 32 for C and H, *n* = 30 for L). Different letters indicate significant differences in the data (after Box–Cox transformation for **A** and **B**) (*p* < 0.05).

To assess the impact of gamma irradiation on fertility, we measured sperm number, sperm viability, and reproductive potential. The number of spermatozoa was not significantly different across conditions (Figure 1A). In contrast, sperm viability was significantly affected by radiation. Compared to C (control), viability was 14.2% lower for L (low dose rate) (*p* < 0.0001) and 20.4% lower for H (High dose rate) (*p* < 0.0001). Viability was also significantly lower in H than in L (*p* = 0.00225), with a decrease of 7.16% (Figure 1B). Reproductive potential was significantly decreased by 14.1% in L compared to C (*p* = 0.035) and by 25.3% in H compared to C (*p* = 0.0004), while there was no significant difference between L and H (*p* = 0.07) (Figure 1C).

### 3.2. Physiological Effects of Ionizing Radiation

#### 3.2.1. Univariate Analyses

**Figure 2:**
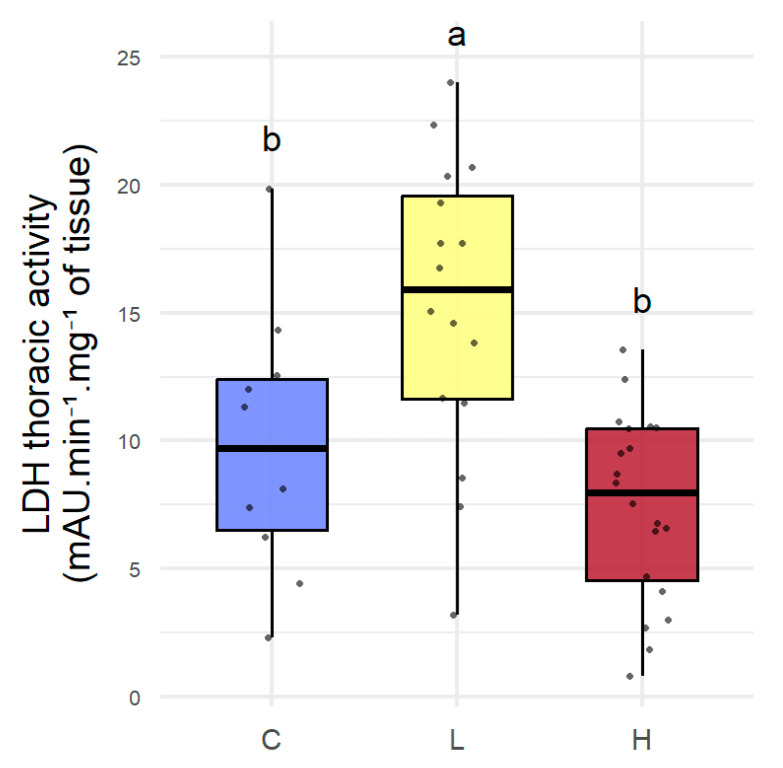
Effects on LDH after 14 days of irradiation. *Apis mellifera* queens irradiated for 14 days with gamma radiation: C (control, non-irradiated), L (irradiated at the low dose of 13 µGy/h), and H (irradiated at the high dose of 3500 µGy/h). Data corresponds to the activity of thoracic LDH expressed in mAU·min^−1^·mg^−1^ of tissue, represented as box plots (*n* = 10 for C, *n* = 16 for L, and *n* = 20 for H). Different letters indicate significant differences between groups (*p* < 0.05).

In order to study the impact of gamma irradiation on the physiology of queens, biomarkers were first analyzed individually. The analysis of the biomarkers revealed no significant effects, except for LDH (Table 2). The activity of LDH in H (*p* = 0.408) did not differ significantly from C. LDH in L is significantly higher by 63.7% compared to C (*p* < 0.02) and by 100.1% compared to H (*p* < 0.001) (Figure 2).

#### 3.2.2. Multivariate Analyses

**Figure 3:**
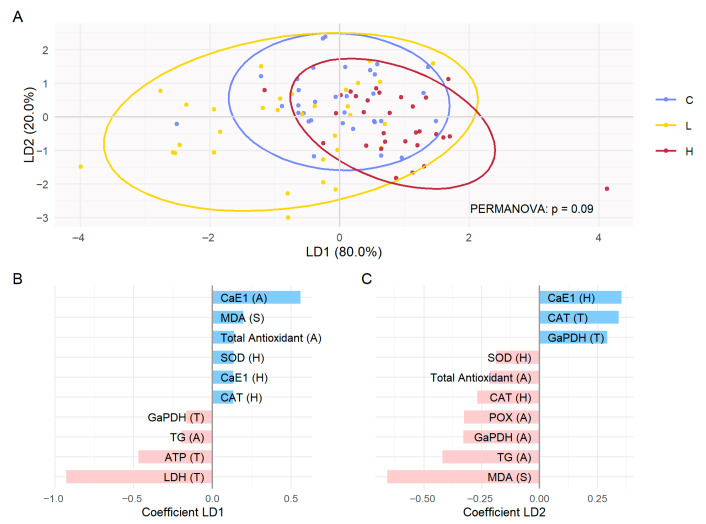
Linear discriminant analysis (LDA) of all biomarkers. *Apis mellifera* queens following exposure to gamma radiation for 14 days: C (control, non-irradiated), L (irradiated at 13 µGy/h), and H (irradiated at 3500 µGy/h). LDA (**A**) represents individuals according to the first two discriminant components (LD1 and LD2), colored according to experimental modality. The ellipses correspond to the 95% confidence intervals for each modality. The *p*-value of the PERMANOVA test is indicated. (**B**) Coefficients of the variables contributing to the LD1 dimension. (**C**) Coefficients of the variables contributing to the LD2 dimension. The LD coefficients indicate the weight and direction of contribution of each biomarker to the separation of groups. Biologically, the biomarkers with the highest coefficients are those that most differentiate C, L, and H. Tissue: H (head), T (thorax), A (abdomen), S (spermatozoa).

Given that only one biomarker was significantly affected by ionizing radiation, multivariate analyses were performed to increase statistical power and detect potential overall physiological patterns. Among all biomarkers, linear discriminant analysis (LDA) indicated that LD1 and LD2 explained 80% and 20% of the discriminant variance between C, L, and H. The 95% ellipses overlapped (PERMANOVA not significant, *p* = 0.09), but trends emerged (Figure 3A). Along LD1, C remained centered, H was shifted towards positive values, while L was shifted towards negative values. The results showed that the positives values of the LD1 axis were mainly associated with CaE1 (abdomen), MDA (spermatozoa), Total antioxidant (abdomen), SOD (head), CaE1 (head) and CAT (head), while the negatives values were driven by LDH (thorax), ATP (thorax), TG (abdomen) and GaPDH (thorax) (Figure 3A,B). On LD2, C showed a slight shift towards positive values, while L and H tended towards negative values. The positive side of LD2 was dominated by CaE1 (head), CAT (head) and GaPDH (head), while the negative side was defined by MDA (spermatozoa), TG (abdomen), GaPDH (abdomen), POx (abdomen), CAT (head), total antioxidant (abdomen) and SOD (head) (Figure 3A,C). Taken together, these patterns indicated that L was more aligned with a “metabolic” profile, whereas H showed a slight tendency toward an “antioxidant/detoxification” profile.

**Figure 4:**
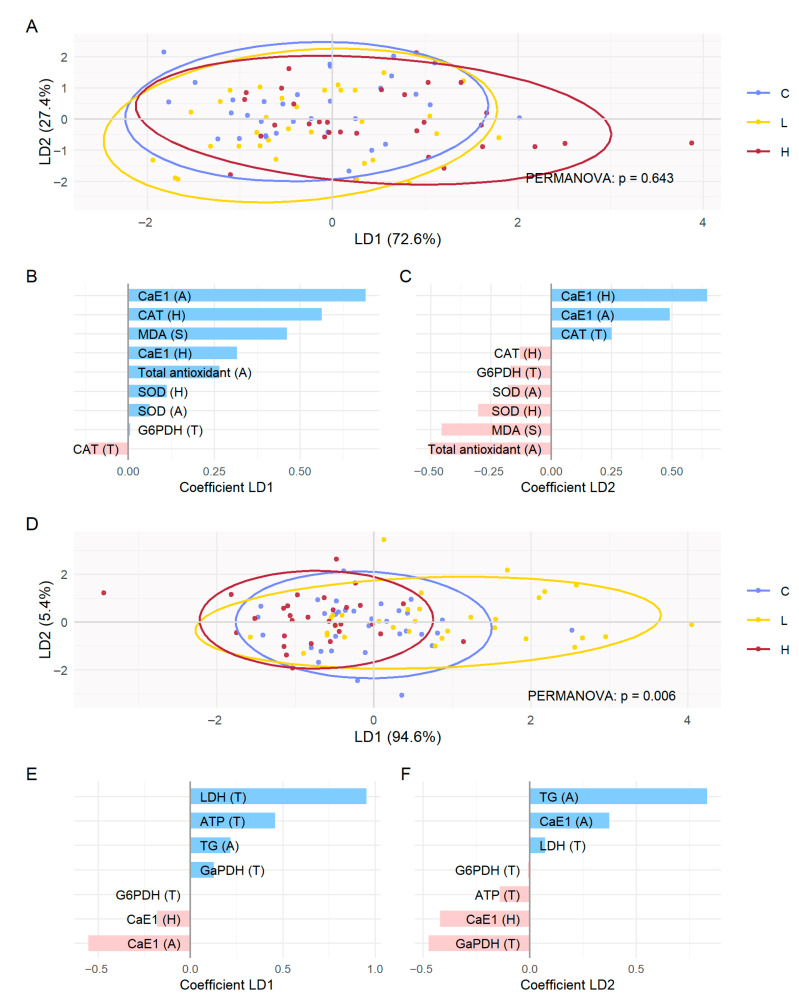
Linear discriminant analysis (LDA) on antioxidant/detoxification and on metabolic biomarkers. *Apis mellifera* queens following exposure to gamma radiation for 14 days: C (control, non-irradiated), L (irradiated at the low dose of 13 µGy/h), and H (irradiated at the high dose of 3500 µGy/h). (**A**) LDA on antioxidant/detoxification biomarkers; (**B**) coefficients of LD1 (antioxidant/detoxification); (**C**) coefficients of LD2 (antioxidant/detoxification). (**D**) LDA on metabolic biomarkers; (**E**) coefficients of LD1 (metabolic); (**F**) coefficients of LD2 (metabolic). In LDA plots, individuals are represented according to the first two discriminant components (LD1 and LD2), colored by modality. The ellipses correspond to the 95% confidence intervals for each modality. The *p*-value of the PERMANOVA test is indicated. In coefficient plots, variables contributing to LD1 or LD2 are shown, and LD coefficients indicate the weight and direction of each biomarker to group separation, with the highest coefficients most differentiating C, L, and H. Tissue: H (head), T (thorax), A (abdomen), S (spermatozoa).

In the LDA restricted to antioxidant and detoxification biomarkers (MDA, CAT, SOD, Total Antioxidant, G6PDH, CaE1), PERMANOVA did not indicate any significant separation between modalities (*p* = 0.643). LD1 explained 72.6% and LD2 27.4% of the variance (Figure 4A). On LD1, H tended to shift toward positive values, driven mainly by CaE1 (abdomen), CAT (head), and MDA (spermatozoa) (Figure 4A,B). On LD2, no clear separation between modalities was observed, the axis being structured positively by CaE1 (head and abdomen) and CAT (thorax), and negatively by Total antioxidant (abdomen) and MDA (spermatozoa) (Figure 4A,C). Consistently, PERMANOVA did not indicate any significant separation between modalities (*p* = 0.643) (Figure 4A).

In the LDA restricted to metabolic biomarkers (G6PDH, GaPDH, ATP, LDH, TG, and CaE1), LD1 explained 94.6% of the variance and LD2 5.4%, discrimination therefore came mainly from LD1 (Figure 4D). On LD1, C remained centered, H and L shifted slightly toward negative values (notable contribution from CaE1 (abdomen and head)), while L was also clearly on the positive side, driven mainly by LDH (thorax), ATP (thorax), TG (Abdomen), and GaPDH (Head) (Figure 4D,E). On LD2, no clear separation between modalities was observed; the axis was mainly structured positively by TG (abdomen) and CaE1 (abdomen) and negatively by GaPDH (head) and CaE1 (head) (Figure 4D,F). The overall PERMANOVA was significant (*p* = 0.006), and pairwise comparisons showed that the difference came exclusively from L vs. H (*p* = 0.003), with no difference detected for C vs. L or C vs. H. Dispersion analysis (betadisper) showed no significant differences in dispersion among modalities, indicating that the significant separation observed between L and H in PERMANOVA is not attributable to differences in within-group variability (Figure 4D and Table 3).

## 4. Discussion

The impacts of a gamma irradiation, at 13 and 3500 µGy/h, on queens of the honeybee *Apis mellifera* L., were determined by focusing on survival, reproductive potential (number and viability of spermatozoa), and a range of biomarkers related to immune defenses, defenses against oxidative stress, energy metabolism, and neural activity.

### 4.1. Effect of Ionizing Radiation on Survival and Fertility

No significant effect of irradiation at 13 or 3500 µGy/h was observed on queen mortality during the 14 days of irradiation. This result is consistent with previous observations showing that worker honeybees exposed to gamma radiation also do not exhibit short-term mortality [36]. Thus, ionizing radiation did not appear to induce short-term mortality in queens at the tested dose rates.

The number of spermatozoa stored in the spermatheca did not differ significantly between control queens and those exposed to 13 or 3500 µGy/h. This could be explained by the fact that the queens were already fertilized before irradiation: the sperm stock is built up and fixed during mating flights and then maintained throughout the queen’s life [3]. However, irradiated queens showed a reduction in sperm viability and a decrease in reproductive potential at 13 and 3500 µGy/h, without a concomitant decrease in the total number of spermatozoa stored. This result may indicate that non-viable spermatozoa are not eliminated from the spermatheca, or that 14 days of irradiation are insufficient to observe this phenomenon.

Both irradiation dose rates caused a decrease in sperm viability, which appeared to be dose-dependent, with a more pronounced effect at 3500 µGy/h than at 13 µGy/h. These observations are consistent with previous reports showing that ionizing radiation can impair sperm viability, as demonstrated, for example, in mice. The reductions observed in mice result from acute 5 Gy (1–1.4 Gy/min) exposures and are therefore not directly comparable to our low-dose chronic conditions, yet both sets of findings highlight that fertility is a process particularly sensitive to ionizing radiation [37,38]. The average viability was 76.4% in the control queens, 66.5% at 13 µGy/h, and 61.6% at 3500 µGy/h. In the study by Pettis et al. [11], queens with sperm viability between 54 and 57% are generally considered to be defective by beekeepers or likely to be replaced by a new queen by the colony. Queens exposed to 3500 µGy/h therefore reached a value (61.6%) close to this critical threshold. Since sperm viability is a key parameter of queen fertility, this decrease in viability suggests that irradiation could impair the reproductive capacity of queens. However, a reduction in sperm viability is not specific to ionizing radiation. It has also been described in response to chemical (pesticides), thermal (cold), or biological (*Nosema ceranae*) stress [39,40]. Although these results were obtained under controlled laboratory exposure, they indicate potential pathways by which ionizing radiation could interact with these factors and accentuate the decay of fertility in queen bees, ultimately compromising the dynamics and survival of colonies.

Reproductive potential, which combines both sperm count and viability, was significantly reduced in queens irradiated at 13 and 3500 µGy/h. This indicates that even relatively low dose rates can reduce the number of viable spermatozoa in the spermatheca. The results also showed that the MDA level in spermatozoa did not differ significantly between exposure modalities. This suggests that the observed decline in reproductive potential is not the result of oxidative stress resulting in lipid peroxidation caused by ionizing radiation. We can hypothesize that a metabolic disturbance may be involved. Although the exposure context differs, Kairo et al. [16] showed that exposure of males to Fipronil increased sperm metabolism and led to a decline in reproductive potential in queens inseminated with this sperm. The analysis of additional antioxidant and metabolic biomarkers in spermatozoa, such as ATP, LDH, CAT, or SOD [16], would allow for a better characterization of the mechanisms affecting their viability. Moreover, the spermathecal fluid, as the immediate environment of the gametes, contains numerous defense and energy-regulating enzymes, such as peroxiredoxins, glutathione peroxidases, or glycolytic enzymes identified by Baer et al. [41], which could represent sensitive indicators of irradiation effects. Although our results were obtained under controlled laboratory conditions, they suggest that exposure at a dose as low as 13 µGy/h could compromise the reproductive success of queen bees, with potential repercussions for the colony. Further investigation, particularly on the performance of entire colonies under real environmental conditions, would be necessary to confirm this hypothesis.

### 4.2. Effect of Ionizing Radiation on Queen Physiology

Multimarker analyses did not indicate any significant differences between the modalities, even when only antioxidant and detoxification biomarkers were taken into account. Similarly, taken individually, the majority of biomarkers did not differ significantly between control queens and those irradiated at 13 or 3500 µGy/h for 14 days. The biomarkers used in this study are recognized as relevant for assessing honeybee health. Indeed, several previous studies have used a battery of biomarkers similar to ours to investigate the effects of various environmental stressors, such as pesticides and/or *Nosema ceranae* infection [40], or ionizing radiation [36]. To our knowledge, this study is the first to apply such a battery of biomarkers specifically to queens. However, effects might have been expected, as observed by Gagnaire et al. [36], who reported significant physiological alterations after 3 to 14 days of irradiation at 0.183 to 2.45 × 10^4^ μGy/h in emerging workers. The contrast between these results and ours suggests that the response to ionizing radiation may be caste-dependent, with queens exhibiting a different sensitivity than workers. This difference could be related to specific protective mechanisms in queens, including greater resistance to oxidative stress and increased protection against pathogens and viruses through individual and social immunity [39,42,43]. Moreover, queens are exclusively fed royal jelly throughout their development, a substance rich in antioxidant and antimicrobial compounds, which could enhance their protection against oxidative stress induced by ionizing radiation [44,45,46]. These characteristics, which contribute to the superior longevity of queens, could also explain why they appear to be less affected by short-term irradiation than workers. Another hypothesis to explain the absence of significant physiological effects is that irradiation occurred after the queen had already been fertilized. It would therefore be relevant to examine the effects of radiation during the nuptial flight, an essential stage of the reproductive cycle that may be more sensitive to irradiation.

Among the biomarkers studied, only thoracic LDH activity differed significantly, with higher activity in queens irradiated at 13 µGy/h than in controls and those irradiated at 3500 µGy/h. Similarly, linear discriminant analysis performed on metabolic biomarkers showed a differentiation between queens exposed to 13 µGy/h and queens irradiated at 3500 µGy/h, mainly driven by an increase in LDH and ATP activities. These results suggest a disruption of energy metabolism induced by low-dose irradiation. LDH plays a central role in maintaining glycolysis by rapidly regenerating NAD^+^ from NADH, allowing for rapid ATP production during high metabolic demand [47]. The observed increase in LDH levels may indicate enhanced mobilization of glycolytic pathways to meet the energy demands of queens irradiated at low dose rates (13 µGy/h), suggesting that such metabolic activation could reflect an energy cost associated with managing radiation-induced stress, as also evidenced by increased ATP production. Although no changes were detected in biomarkers of oxidative stress or immunity, it is possible that this reallocation of resources to energy metabolism limits the energy available for costly processes such as reproduction or cell maintenance, which could contribute to the short-term alterations in fertility observed and possibly influence longevity in the longer term. One might have expected the effect observed on metabolism at 13 µGy/h to be amplified at 3500 µGy/h. However, no significant effect was observed, suggesting a non-proportional response to ionizing radiation. Gagnaire et al. [36] also described similar responses in emerging workers exposed to a gamma radiation gradient, particularly for carboxylesterases. Thus, low-dose exposures could induce specific metabolic adjustments, distinct from those triggered by higher dose rates. Such non-proportional dose–response patterns have also been described in radiobiology, where biological effects are not always proportional to dose or dose rate, especially at low exposure levels [13].

## 5. Conclusions

This study aimed to evaluate the effects of gamma irradiation on the mortality, fertility, and physiology of queen honeybees. Irradiation did not alter queen survival or the number of sperm stored in the spermatheca, but it did reduce sperm viability and reproductive potential at 13 and 3500 µGy/h. Most physiological biomarkers showed no differences between the modalities, except for a disturbance in energy metabolism at low dose rates. Although the reduction in sperm viability remained above the threshold generally associated with defective queens in beekeeping practice, it approached this critical level at the highest exposure rate tested. These results suggest that ionizing radiation did not induce lethality under the tested modalities, but significantly reduced sperm viability, reproductive potential, and metabolism of queen honeybees, with potential consequences for colony functioning that deserve further investigation under realistic environmental exposure scenarios.

## Figures and Tables

**Table 1:** Biomarkers used and their targeted biological functions in tissues.

Biomarker	Tissue	Functions
AChE	Head	Neural activity
MDA	Spermatozoa	Oxidative damages/Antioxidant defenses
CAT	Head/Thorax	Antioxidant defenses/Detoxification
SOD	Head/Abdomen	Antioxidant defenses
Total antioxidant	Abdomen	Antioxidant defenses
G6PDH	Thorax	Antioxidant defenses/Metabolism
GaPDH	Abdomen/Thorax	Metabolism/Antioxidant defenses
ATP	Thorax	Metabolism
LDH	Thorax	Metabolism
TG	Abdomen	Metabolism
CaE1	Head/Abdomen	Detoxification/Metabolism/Immunity
POx	Abdomen	Immunity

**Table 2:** Effects of ionizing radiation on biomarkers. The values are the means ± standard deviation (SD) per biomarker with the tissue analyzed and unit (AU: absorbance unit; LI: luminescence intensity), in queens irradiated for 14 days according to three modalities (0.1, 13, and 3500 µGy/h). The “Data” column specifies the data on which the statistical tests were performed: “Raw” = values without transformation; “Box–Cox” = values after Box–Cox transformation. The “statistical test” column indicates the overall test comparing the three modalities: ANOVA when normality and homogeneity of variances are satisfied, otherwise Kruskal–Wallis (on raw data). The *p*-value reported is that of this overall test.

Biomarker and Targeted Tissue (Unit)	Physiological Marker Responses (Mean ± SD) *			Statistical Tests		
	Control (0.1 µGy/h)	Low Dose Rate (13 µGy/h)	High Dose Rate (3500 µGy/h)	Data	Tests	*p*-Value
Head AChE (mAU·min^−1^·mg^−1^ of tissue)	85.5 ± 11.8	85.6 ± 14.6	83.4 ± 21.4	Raw	Kruskal-Wallis	0.842
Head CAT (mAU·min^−1^·mg^−1^ of tissue)	16 ± 6.9	16.2 ± 8.4	19.3 ± 10.3	Box-Cox	ANOVA	0.31
Head SOD (mAU·min^−1^·mg^−1^ of tissue)	2.6 ± 0.8	2.6 ± 0.7	2.8 ± 0.4	Box-Cox	ANOVA	0.813
Head CaE1 (mAU·min^−1^·mg^−1^ of tissue)	0.7 ± 0.1	0.7 ± 0.09	0.7 ± 0.1	Box-Cox	ANOVA	0.555
Abdomen SOD (mAU·min^−1^·mg^−1^ of tissue)	4.5 ± 1.1	4.6 ± 1.2	4.5 ± 1.2	Raw	ANOVA	0.869
Abdomen Total Antioxidant (mMol Trolox)	2.1 ± 0.2	2.2 ± 0.2	2.2 ± 0.2	Raw	ANOVA	0.375
Abdomen GaPDH (mAU·min^−1^·mg^−1^ of tissue)	392 ± 55.4	409.4 ± 111.5	412.8 ± 69.4	Raw	Kruskal-Wallis	0.772
Abdomen CaE1 (mAU·min^−1^·mg^−1^ of tissue)	1493.3 ± 121.6	1460.3 ± 136.8	1535.8 ± 115.2	Raw	ANOVA	0.067
Abdomen POx (mAU·min^−1^·mg^−1^ of tissue)	3.6 ± 2.3	4.6 ± 2.8	3.8 ± 2.3	Box-Cox	ANOVA	0.286
Abdomen TG (mg/µL)	12.3 ± 4.4	13.5 ± 5.9	13.4 ± 2.7	Box-Cox	ANOVA	0.387
Thorax CAT (mAU·min^−1^·mg^−1^ of tissue)	6.1 ± 2.7	6.1 ± 2.9	6.1 ± 3.5	Box-Cox	ANOVA	0.989
Thorax G6PDH (mAU·min^−1^·mg^−1^ of tissue)	9.6 ± 1.4	9.6 ± 2.5	9.5 ± 2.1	Box-Cox	ANOVA	0.853
Thorax GaPDH (mAU·min^−1^·mg^−1^ of tissue)	566.6 ± 110.5	545.1 ± 109.6	554.7 ± 142	Raw	ANOVA	0.818
Thorax ATP (LI × 10^6^·mg^−1^ of tissue)	2.8 ± 0.8	3 ± 0.9	2.6 ± 0.7	Box-Cox	ANOVA	0.225
Thorax LDH (mAU·min^−1^·mg^−1^ of tissue)	9.9 ± 5.2	15.3 ± 5.7	7.5 ± 3.6	Raw	ANOVA	<0.0001
Spermatozoa MDA (µMol)	0.2 ± 0.2	0.3 ± 0.2	0.3 ± 0.2	Box-Cox	ANOVA	0.249

* Head AChE; *n* = 32 (C, L), *n* = 31 (H); Head CAT; *n* = 31 (C, H), *n* = 29 (L); Head SOD; *n* = 23 (C), *n* = 22 (L), *n* = 21 (H); Head CaE1; *n* = 31 (C), *n* = 25 (L), *n* = 27 (H); Abdomen SOD; *n* = 33 (C), *n* = 32 (L, H); Abdomen Total Antioxidant; *n* = 28 (C), *n* = 30 (L), *n* = 29 (H); Abdomen GaPDH; *n* = 33 (C), *n* = 32 (L), *n* = 31 (H); Abdomen CaE1; *n* = 33 (C), *n* = 31 (L, H); Abdomen POx; *n* = 33 (C), *n* = 31 (L,H); Abdomen TG; *n* = 28 (C), *n* = 30 (L), *n* = 29 (H); Thorax CAT; *n* = 30 (C, H), *n* = 28 (L); Thorax G6PDH; *n* = 30 (C, L), *n* = 29 (H); Thorax GaPDH; *n* = 24 (C), *n* = 28 (L), *n* = 27 (H); Thorax ATP; *n* = 30 (C, L, H); Thorax LDH; *n* = 10 (C), *n* = 16 (L), *n* = 20 (H); Spermatozoa MDA; *n* = 19 (C), *n* = 18 (L), *n* = 17 (H).

**Table 3:** Effects of ionizing radiation on multivariate metabolic profiles. Results of pairwise PERMANOVA and dispersion tests (betadisper) performed on metabolic biomarkers (G6PDH, GaPDH, ATP, LDH, TG, and CaE1) measured in queens after 14 days of exposure (C = control, non-irradiated; L = 13 µGy/h; H = 3500 µGy/h). The values shown correspond to *p*-values.

Analysis	*p*-Values		
	C vs. L	C vs. H	L vs. H
Pairwise PERMANOVA	0.139	0.379	0.003
Dispersion (betadisper)	0.157	0.94	0.304

## Data Availability

The original contributions presented in this study are included in the article/Supplementary Materials. Further inquiries can be directed to the corresponding author.

## Supplementary Materials

The following supporting information can be downloaded at: https://www.mdpi.com/article/10.3390/toxics13121057/s1, Table S1: Raw data of mortality; Table S2: Raw data of fertility; Table S3: Raw data of biomarkers.

## Abbreviations

The following abbreviations are used in this manuscript:

MICADO’Lab	Chronic irradiation method for establishing dose–effect relationships in the laboratory
