# Supplementary material for: Effects of Ionizing Radiation on Apis mellifera L. Queens
Source: Toxics. 2025 Dec 5;13(12):1057. doi: 10.3390/toxics13121057 (PMC12737656; doi:10.3390/toxics13121057)
Supplement: Supplementary file 1 [file toxics-13-01057-s001.zip › toxics-3976533-Supplementary Materials.pdf]

Supplementary Materials

Table S1. Raw data of mortality. C: Control queen bees, neither irradiated nor infected. L: Queen bees irradiated at 13 μGy/h. H: Queen bees irradiated at 3500 μGy/h. The data shows the number of queens alive and dead at the end of the 14-day experiment.

Table S2. Raw data of fertility. C: Control queen bees, neither irradiated nor infected. L: Queen bees irradiated at 13 μGy/h. H: Queen bees irradiated at 3500 μGy/h. NA: not available.

Table S3. Raw data of biomarkers. C: Control queen bees, neither irradiated nor infected. L: Queen bees irradiated at 13 μGy/h. H: Queen bees irradiated at 3500 μGy/h. NA: not available. Biomarkers measured in the (A): Abdomen, (T): Thorax, (H): Head, (S): Spermatozoa. The data are expressed in milli Absorbance Units.min^{-1}.mg^{-1} of tissue for SOD, GaPDH, Pox, CaE1, LDH, G6PDH, CAT and AChE; in mMol Trolox for Total antioxidant; in mg/μL for TG; in Luminescence Intensity.mg^{-1} of tissue for ATP and in μMol for MDA.

Table S1. Raw data of mortality. C: Control queen bees, neither irradiated nor infected. L: Queen bees irradiated at 13 µGy/h. H: Queen bees irradiated at 3500 µGy/h. The data shows the number of queens alive and dead at the end of the 14-day experiment.

	Modalities		
	C	T	H
**Alive queens**	65	60	60
**Dead queens**	0	0	0

Table S2. Raw data of fertility. C: Control queen bees, neither irradiated nor infected. L: Queen bees irradiated at 13 µGy/h. H: Queen bees irradiated at 3500 µGy/h. NA: not available.

Modalities	Number of spermatozoa (10^6 / µL semen)	Sperm viability (%)	Reproductive potential (number of live spermatozoa x10^6/µL semen)
**C**	12.71	76.33	9.95
**C**	19.38	78.33	16.28
**C**	7.71	84	5.37
**C**	17.71	69.67	14.82
**C**	22.29	83.67	15.83
**C**	10.63	71	8.25
**C**	5.73	77.67	3.95
**C**	7.19	69	5.87
**C**	12.19	81.67	9.18
**C**	10	75.33	8.37
**C**	7.92	83.67	6.78
**C**	14.06	85.67	10.59
**C**	9.17	75.33	6.94
**C**	5.73	75.67	4.26
**C**	10.1	74.33	7.21
**C**	12.19	71.33	8.82
**C**	5.31	72.33	4.18
**C**	8.96	78.67	7.05
**C**	8.96	78.67	6
**C**	12.19	67	9.83
**C**	10.1	80.67	7.38
**C**	6.46	73	5.23
**C**	20	81	15.40
**C**	9.69	77	7.39
**C**	12.71	70	8.9
**C**	7.29	68.67	5.01
**C**	8.65	85.33	7.38
**C**	10.73	78.67	8.44
**C**	12.92	86.67	11.19
**C**	16.35	70.67	11.56
**C**	10.21	71.33	7.28
**C**	8.85	73.67	6.52

**L**	11.88	68.67	8.15
**L**	9.06	65	5.89
**L**	7.92	65.33	5.17
**L**	11.46	67	6.3
**L**	14.48	55	11.15
**L**	10.52	77	7.12
**L**	7.4	67.67	4.59
**L**	16.88	62	12.66
**L**	13.96	75	8.51
**L**	9.9	61	6.2
**L**	16.56	62.67	12.75
**L**	4.48	77	2.64
**L**	11.15	59	6.98
**L**	10.83	62.67	6.72
**L**	10.21	62	6.98
**L**	10.52	68.33	6.38
**L**	14.58	60.67	9.33
**L**	8.54	64	6.06
**L**	11.88	71	7.48
**L**	9.48	63	6.7
**L**	10	61.33	3.26
**L**	5.31	72.67	5.6
**L**	7.71	62	4.71
**L**	7.60	78.33	5.47
**L**	6.98	63	5.05
**L**	8.02	64.67	7.07
**L**	10.94	65.33	8.44
**L**	12.92	68	5.74
**L**	8.44	66.67	5.28
**L**	7.92	77.67	4.69
**L**	6.04	NA	NA
**H**	11.15	56.67	6.32
**H**	11.04	59	6.51
**H**	8.44	66	5.57
**H**	8.85	60	5.31
**H**	7.6	55.67	4.23
**H**	13.65	58.67	8.01
**H**	17.4	57.67	10.03
**H**	9.17	55	5.04
**H**	16.35	60.33	9.87
**H**	15.73	53.67	8.44
**H**	7.71	71	5.47
**H**	8.33	61.33	5.11
**H**	4.06	67.33	2.74
**H**	4.79	59.33	2.84
**H**	11.04	55	6.07

**H**	5.42	55	2.98
**H**	5.63	68	3.83
**H**	6.98	53	3.7
**H**	11.98	66.67	7.99
**H**	6.98	60.67	4.23
**H**	11.46	60	6.88
**H**	11.77	65.67	7.73
**H**	9.06	75.33	6.83
**H**	9.27	58.67	5.44
**H**	4.38	66.33	2.9
**H**	8.13	69.67	5.66
**H**	6.15	64.67	3.97
**H**	8.75	61.67	5.4
**H**	13.02	54	7.03
**H**	11.35	65	7.38
**H**	10.38	64	6.64
**H**	6.15	67	4.12

Table S3. Raw data of biomarkers. T: Control queen bees, neither irradiated nor infected. L: Queen bees irradiated at 13 µGy/h. H: Queen bees irradiated at 3500 µGy/h. NA: not available. Biomarkers measured in the (A): Abdomen, (T): Thorax, (H): Head, (S): Spermatozoa. The data are expressed in milli Absorbance Units.min^{-1}.mg^{-1} of tissue for SOD, GaPDH, Pox, CaE1, LDH, G6PDH, CAT and AChE; in mMol Trolox for Total antioxidant; in mg/µL for TG; in Luminescence Intensity.mg^{-1} of tissue for ATP and in µMol for MDA.

**Modalities**	**SOD (A)**	**GaPDH (A)**	**TG (A)**	**Total antioxidant (A)**	**POx (A)**	**CaE1 (A)**	**LDH (T)**	**GaPDH (T)**	**G6PDH (T)**	**CAT (T)**	**ATP (T)**	**CaE1 (H)**	**CAT (H)**	**SOD (H)**	**AChE (H)**	**MDA (S)**
**C**	4.99	430.52	10.56	1.87	3.09	1394.74	7.39	669.52	8.48	6.54	2197849.46	0.68	26.88	3.32	86.94	0.01
**C**	7.49	168.9	12.87	1.92	2.29	1407.27	2.31	566.05	9.82	4.87	3070967.74	0.68	29.93	2.78	86.14	0.04
**C**	4.63	435.28	11.84	2.24	4.76	1511.47	4.41	429.99	7.76	6.08	3221505.38	0.68	13.82	2.98	102.75	0.02
**C**	4.86	375.05	10.89	2.28	2.6	1351.88	12.56	486.69	9.78	5.17	2649462.37	0.68	14.57	2.13	91.04	0.62
**C**	4.99	407.34	11.01	2.19	4.66	1379.58	19.87	511.27	12.03	9.23	2769892.47	0.67	7.58	3.05	67.91	0.25
**C**	5.42	391.44	12.87	1.89	2.07	1403.98	6.24	599.29	9.6	4.24	2980645.16	0.68	8.88	3.12	88.11	0.02
**C**	3.62	439.63	15.11	1.94	5.04	1705.35	11.33	581.5	10.96	5.83	2649462.37	0.68	12.49	2.74	110.8	0.27
**C**	3.65	402.37	10.77	2.52	5.22	1521.36	12	528.36	8.77	6.94	2950537.63	0.54	15.56	2.20	48.23	0.01
**C**	2.78	453.29	14.82	2.4	2.09	1710.62	8.12	846.97	12.19	4.5	3070967.74	0.51	14.27	2.84	83.14	0.16
**C**	2.67	416.03	9.22	2.19	3.87	1546.42	14.34	446.38	6.66	7.82	2438709.68	0.58	10.73	3.09	90.23	0.03
**C**	2.62	396.45	7.57	2.33	2.88	1682.67	NA	529.41	8.51	4.96	4266666.67	0.57	8.26	2.17	89.36	0.04
**C**	4.68	404.96	3.66	1.78	0.34	1340.67	NA	514.38	8.94	5.65	2533333.33	0.77	12.55	2.93	82.58	0.16
**C**	5.53	387.23	20.33	2.21	2.17	1381.33	NA	594.12	8.59	3.66	2166666.67	0.7	18.87	2.4	82.78	0.05
**C**	4.7	417.02	5.51	2.29	2.74	1390	NA	426.47	8.82	6.43	2833333.33	0.7	10.47	2.31	99.42	0.33
**C**	5.18	378.72	4.69	2.24	2.22	1338.67	NA	768.63	11.45	8.06	2666666.67	0.7	14.62	3.02	81.7	0.64
**C**	2.13	380.14	18.89	2.3	7.43	1476.67	NA	622.13	9.88	9.16	2900000	0.84	15.11	2.45	77.25	0.15
**C**	4.43	397.16	19.09	2.25	8.60	1498.67	NA	437.15	9.24	6.27	2433333.33	0.88	13.4	3.19	90.78	0.33
**C**	4.37	377.3	14.98	2.17	7.48	1508	NA	516.24	9.12	4.73	2366666.67	0.72	15.18	2.34	82.06	0.2
**C**	6.95	399.29	19.09	1.62	0.44	1748.67	NA	634.88	9.25	4.85	2966666.67	0.55	40.78	4.35	83.83	0.12
**C**	4.76	381.56	11.48	2.32	1.38	1567.33	NA	519.8	12.24	7.45	2866666.67	0.59	19.88	3.09	96.91	NA
**C**	4.16	436.12	11.87	2.12	3.89	1475.65	NA	543.35	10.88	2.61	1742857.14	0.58	16.16	0.88	73.71	NA
**C**	4.62	451.32	8.23	2.02	1.84	1320.02	NA	435.61	8.3	12.22	1657142.86	0.79	11.08	1.2	62.1	NA
**C**	4.99	373.07	10.44	2.11	0.74	1450.29	NA	675.04	8.81	0.24	3542857.14	0.61	11.85	0.93	83.99	NA
**C**	3.65	436.58	14.08	2.26	1.87	1530.99	NA	715.2	11.14	2.22	2514285.71	0.98	13.3	NA	87.65	NA

**C**	4.39	430.34	20.05	2.12	4.2	1524.07	NA	NA	10.06	9.51	3114285.71	0.82	11.35	NA	83.16	NA
**C**	5.08	444.51	10.7	2.2	2.93	1632.44	NA	NA	9.5	10.6	5142857.14	0.56	18.66	NA	87.96	NA
**C**	5.49	318.08	10.7	1.88	9.68	1589.21	NA	NA	8.11	3.49	4342857.14	0.69	21.28	NA	80.68	NA
**C**	3.46	369.1	13.17	2.23	4.19	1437.03	NA	NA	9.22	9.67	2257142.86	0.64	20.42	NA	99.73	NA
**C**	4.06	268.75	NA	NA	2.77	1479.69	NA	NA	9.22	6.92	1771428.57	0.62	21.97	NA	99.42	NA
**C**	4.09	379.29	NA	NA	3.63	1689.71	NA	NA	10.8	3.73	1914285.71	0.67	16.44	NA	90.59	NA
**C**	4.8	409.66	NA	NA	1.37	1549.83	NA	NA	NA	NA	NA	0.93	9.58	NA	83.03	NA
**C**	5.09	353.51	NA	NA	7.03	1389.74	NA	NA	NA	NA	NA	NA	NA	NA	82.96	NA
**C**	4.51	425.48	NA	NA	2.24	1343.79	NA	NA	NA	NA	NA	NA	NA	NA	NA	NA
**L**	5.36	898.63	9.03	2.23	6.11	1603.79	20.69	580.8	8.65	9.7	2438709.68	0.66	26.26	3.48	97.92	0.42
**L**	5.9	314.45	10.27	2.26	7.19	1282.64	8.54	484.58	7.36	13.78	3703225.81	0.67	17.3	2.6	82.46	0.82
**L**	7.92	355.68	13.08	2.22	3.31	1396.72	15.08	460.7	10.87	4.09	3131182.80	0.69	16.7	2.24	87.97	0.65
**L**	4.23	345.74	10.52	2.51	2.34	1284.62	11.7	405.46	7.5	7.9	2920430.11	0.53	13.37	2.24	93.09	0.21
**L**	5.80	392.44	17.01	1.88	4.97	1530.59	19.31	613.34	7.43	6.15	2559139.78	0.64	11.55	3.33	73.33	0.01
**L**	3.27	293.58	14.11	2.04	4.29	1198.23	17.73	548.49	10.39	4.6	2769892.47	0.7	39.48	2.41	98.45	0.46
**L**	3.09	380.68	10.97	2.16	9.28	1582.03	11.49	620.36	10.61	7.16	2920430.11	0.58	41.7	2.8	57.96	0
**L**	2.4	447.91	15.27	2.34	6.63	1549.06	20.36	492.17	8.42	4.09	3191397.85	0.55	20.74	2.91	81.82	0.01
**L**	2.01	574.58	13.49	2.3	4.35	1560.93	13.84	509.87	9.38	9.27	3010752.69	0.67	14.89	2.91	68.65	0.6
**L**	6.31	576.24	10.48	2.31	6.11	1572.8	22.37	665.77	7.67	4.53	3221505.38	0.69	12.17	3.09	64.69	0.14
**L**	5.11	374.47	5.51	1.73	2.49	1438.67	24.01	501.96	18	3.1	2166666.67	0.68	9.02	2.72	132.34	0.13
**L**	4.47	389.36	6.54	2.33	2.87	1471.33	7.45	476.47	15.06	6.08	3233333.33	0.72	7.02	1.88	98.16	0.19
**L**	4.61	412.06	19.51	2.3	2.57	1406.67	16.76	581.7	10.75	5.33	2600000	0.62	14.78	3.02	83.55	0.2
**L**	5.04	387.94	15.19	2.29	13.19	1350.67	17.75	673.53	8.63	7.14	4833333.33	0.53	5.74	3.11	94.75	0.27
**L**	5.21	346.81	33.5	2.4	7.46	1338.67	3.19	485.29	8.14	5.55	3300000	0.71	6.94	2.72	71.09	0.25
**L**	4.40	410.64	24.24	2.13	3.23	1474.67	14.61	605.88	9.25	5.88	2733333.33	0.64	23.7	2.15	95.49	0.08
**L**	4.26	398.58	11.07	2.32	4.31	1488.67	NA	644.44	8.61	4.51	2800000	0.56	15.88	2.31	78.87	0.11
**L**	4.28	406.38	12.51	2.38	1.62	1514.67	NA	507.44	7	8.63	2666666.67	0.85	19.76	3.11	73.62	0.03
**L**	2.91	382.27	15.8	2.74	4.91	1357.33	NA	384.63	8.12	5.78	2900000	0.75	20.7	3.19	71.49	NA
**L**	5.22	401.42	12.78	2.27	5.76	1441.64	NA	543.82	11.39	11.06	1514285.71	0.65	9.27	3.85	77.02	NA
**L**	5.22	415.03	9.14	2.12	6.17	1423.2	NA	514.78	6.54	0.84	1628571.43	0.65	10.03	0.99	73.8	NA

**L**	4.71	384.41	25.51	2.26	2.32	1315.98	NA	552.62	9.63	1.6	2085714.29	0.86	12.85	0.99	116.76	NA
**L**	6.09	444.51	11.48	2.11	4.05	1445.68	NA	352.19	12.18	1.86	2314285.71	0.73	9.86	NA	82.92	NA
**L**	5.25	346.99	8.36	2	1.67	1224.33	NA	330.18	8.1	3.02	4685714.29	0.6	16.42	NA	83.93	NA
**L**	4.23	416.16	10.57	1.85	1.47	1637.05	NA	599.19	12.3	8.33	2942857.14	0.55	12.78	NA	88.67	NA
**L**	4.34	379.88	12.65	2.06	0.37	1615.15	NA	614.80	8.75	7.96	5200000	NA	13.27	NA	88.34	NA
**L**	4.11	433.17	15.12	2.17	4.33	1614.57	NA	673.5	7.46	7.56	4171428.57	NA	13.94	NA	98.08	NA
**L**	5.09	312.97	10.7	2.14	2.45	1781.16	NA	838.78	7.84	5.07	1914285.71	NA	16.26	NA	89.82	NA
**L**	4.18	382.15	10.96	1.93	1.12	1544.25	NA	NA	8.75	NA	2542857.14	NA	18.46	NA	82.44	NA
**L**	4.49	496.68	8.36	1.91	4.66	1616.88	NA	NA	12.42	NA	3514285.71	NA	NA	NA	77.74	NA
**L**	4.22	260.98	NA	NA	9.68	1380.65	NA	NA	NA	NA	NA	NA	NA	NA	89.58	NA
**L**	4.64	339.28	NA	NA	NA	1285.21	NA	NA	NA	NA	NA	NA	NA	NA	83.93	NA
**H**	5.11	373.56	15.48	2.09	5.04	1423.76	6.8	491.61	8.24	4.24	2800000	0.67	18.86	3.05	91.33	0.28
**H**	4.96	336.8	10.77	2.14	5.30	1551.03	1.85	567.45	9.12	6.2	4756989.25	0.62	7.36	2.7	122.89	0.42
**H**	3.1	435.9	9.94	2.25	2.14	1731.73	10.55	561.48	10.35	5.03	3040860.22	0.67	12.57	2.7	96.16	0.47
**H**	4.4	366.77	15.19	1.91	3.8	1615	4.68	471.38	7.01	4.17	2860215.05	0.65	13.45	2.7	38.2	0.04
**H**	4.54	376.29	13.16	2.3	3.51	1565.54	10.51	438.23	13.41	12.95	2378494.62	0.63	9.73	2.62	29.27	0.01
**H**	3.1	437.15	12.75	2.13	9.02	1458.05	4.1	529.53	8.65	13.17	2438709.68	0.62	22.3	3.12	82.92	0.35
**H**	3.6	379.02	9.07	2.38	8.27	1608.41	10.47	544.98	7.8	6.45	3010752.69	0.62	31.69	2.13	114.02	0.27
**H**	2.47	439.63	14.03	2.03	5.97	1616.98	6.48	643.3	8.5	8.09	2800000	0.55	17.02	2.55	95.14	0.09
**H**	1.99	564.65	15.73	2.67	2.39	1816.14	0.82	622.7	10.52	2.89	2739784.95	0.63	41.48	3.05	65.28	0.2
**H**	2.09	655.72	13.45	2.31	4.85	1734.36	8.69	647.06	6.57	9.48	2980645.16	0.62	23.79	2.62	34.1	0.2
**H**	5.74	372.34	16.83	1.75	1	1460.67	2.7	754.90	9.53	7.71	2166666.67	0.72	46.74	2.41	91.7	0.12
**H**	3.69	393.62	10.86	2.44	1.46	1500	9.53	543.14	10.71	3.53	2866666.67	0.78	14.15	2.53	102.77	0.36
**H**	5.46	397.16	11.48	2.32	2.71	1448.67	12.39	641.18	7.97	3.69	3566666.67	0.66	10.64	3.11	85.25	0.06
**H**	5.74	358.87	11.07	2.24	8.2	1420.67	6.59	564.71	11.22	4.45	2633333.33	0.83	9.56	2.84	88.09	0.12
**H**	5.15	361.7	17.04	2.33	7.18	1392	3.01	796.08	5.06	6.73	3000000	0.72	17.63	2.8	72.06	0.75
**H**	4.41	394.33	12.3	2.44	2.06	1503.33	8.37	774.51	8.33	5.65	2300000	0.88	9.62	3.57	76.6	0.76
**H**	5.18	402.84	9.42	2.11	2.54	1654.00	7.55	764.71	7.41	4.8	3000000	0.64	9.96	3.02	100.71	0.72
**H**	7.34	397.87	11.89	2.34	7.63	1608.67	13.57	567.65	13.07	10.02	3100000	0.8	12.13	2.45	100.21	NA
**H**	6.45	382.98	15.19	2.63	2.88	1636	10.76	312.8	10.27	3.25	3733333.33	0.7	33.62	1.86	86.81	NA

**H**	5.6	377.3	18.48	2.27	3.3	1462	9.73	323.23	9.14	14.94	1733333.33	0.57	42.13	3.55	61.49	NA
**H**	3.74	364.46	13.43	2.25	9.35	1359.79	NA	357.6	9.02	4.78	2166666.67	0.54	34.68	0.91	85.67	NA
**H**	4.62	470.59	11.35	2.2	2.41	1287.16	NA	393.9	8.05	2.61	828571.43	0.57	21.42	NA	104.59	NA
**H**	5.08	492.14	11.87	2.23	1.41	1551.74	NA	368.41	13.41	8.64	1942857.14	0.63	19.85	NA	82.51	NA
**H**	5.17	435.44	16.55	2.19	1.79	1556.93	NA	564.59	10.78	2.96	2800000	0.71	23.69	NA	70.01	NA
**H**	4.57	453.47	13.95	2.21	2.65	1433	NA	280.37	10.66	1.24	1857142.86	0.92	15.06	NA	88.98	NA
**H**	3.05	393.49	17.33	2.06	2.92	1581.14	NA	607.07	9.98	7.1	2171428.57	0.85	20.21	NA	75.99	NA
**H**	5.26	353.80	12.52	2.02	3.5	1537.91	NA	646.46	11.82	3.11	2200000	0.64	13.56	NA	90.12	NA
**H**	4.48	433.74	17.07	2.27	3.9	1486.61	NA	NA	8.37	2.64	3057142.86	NA	16.73	NA	86.41	NA
**H**	4.89	469.46	10.7	1.77	1.9	1442.8	NA	NA	11.41	8.71	1914285.71	NA	18.33	NA	82.1	NA
**H**	3.11	299.45	NA	NA	4.23	1454.89	NA	NA	NA	2.49	1628571.43	NA	10.7	NA	92.45	NA
**H**	4.52	378.82	NA	NA	1.23	NA	NA	NA	NA	NA	NA	NA	16.14	NA	94.04	NA
**H**	4.47	NA	NA	NA	NA	NA	NA	NA	NA	NA	NA	NA	NA	NA	NA	NA
